# Carglumic acid and mesalazine as potential anti-mycobacterial agents: a spectroscopic study for repurposing drugs against *Mycobacterium tuberculosis* targeting its essential enzyme ThyX

**DOI:** 10.1128/spectrum.02486-24

**Published:** 2025-10-13

**Authors:** Sana Tanweer, Meetu Agarwal, Kunal Malik, Rahul Sharma, Shivani A. Muthu, Md Abrar Siddiquee, Khushbu Sharma, Isha Pahuja, Waseem Ali, Abhinav Grover, Ved Prakash Dwivedi, Basir Ahmad, Sonam Grover

**Affiliations:** 1 Department of Molecular Medicine, Jamia Hamdard, New Delhi, India; 2 Centre for Interdisciplinary Research in Basic Sciences, Jamia Millia Islamia 28849, New Delhi, India; 3 Immunobiology Lab, International Centre for Genetic Engineering and Biotechnology, New Delhi, India; 4 School of Biotechnology, Jawaharlal Nehru University 28754, New Delhi, India; 5 Department of Medical Elementology and Toxicology, Jamia Hamdard, New Delhi, India; Weill Cornell Medicine, New York, New York, USA

**Keywords:** *M.tb*, thymidylate synthase, tryptophan quenching, spectroscopy, FACS, *in vitro*, *ex vivo*, H_37_Rv, macrophages, propidium iodide (PI) staining

## Abstract

*Mycobacterium tuberculosis (M.tb*) needs a key enzyme called ThyX to make thymidylate, which is essential for DNA replication and cell survival. In our previous study, carglumic acid (CGA) and mesalazine (MSZ) emerged as promising candidates from a Food and Drug Administration-approved drug library, selected through *in silico* screening. Our current investigation delves into the impact of CGA and MSZ on ThyX’s biophysical properties. Utilizing fluorescence quenching, thermal, chemical denaturation, characterization, and circular dichroism spectroscopy, we probed the interaction between ThyX and the drugs. Our results confirm that both CGA and MSZ effectively quench ThyX’s intrinsic fluorescence via a static quenching mechanism, leading to structural alterations in the protein. In subsequent *in vitro* and *ex vivo* studies, we determined that MSZ and CGA exhibit minimum inhibitory concentrations of 6.25 and 3.12 µg/mL, respectively, against *M.tb*. Notably, the survival of *M.tb* within RAW macrophages significantly decreased upon treatment with CGA and MSZ compared to untreated controls. In summary, our findings support the potential repurposing of CGA and MSZ as anti-tuberculosis (TB) drugs. Further validation in animal and clinical models is essential to assess their suitability for TB treatment.

**IMPORTANCE:**

ThyX (*Rv2754c*), flavin-dependent thymidylate synthase, is a crucial enzyme required by *Mycobacterium tuberculosis* for DNA replication and RNA maturation, making it a potential drug target to explore novel anti-tuberculosis (TB) treatments. Given the essentiality of ThyX, it was screened against Food and Drug Administration-approved drugs using molecular docking screening, and carglumic acid (CGA) and mesalazine (MSZ) were selected as potential inhibitors. To validate and explore their anti-mycobacterial potential, molecular dynamic simulation of these drugs in the presence of ThyX was carried out, and these studies were validated using *in vitro* biophysical characterization to establish their binding kinetics and effects of these drugs on the stability and structural changes of ThyX. Lastly, *in vitro* and *ex vivo* anti-mycobacterial activity of CGA and MSZ establish them as probable candidates for management of TB.

## INTRODUCTION

As per the World Health Organization Global Tuberculosis Report 2024, which was based on data from 2023, tuberculosis (TB) is predicted to have killed 1.25 million people worldwide and has surpassed COVID-19 to become the world’s most infectious agent, leading to mortality (1). Drug-resistant TB is of major concern, and understanding the link between antibiotic resistance and virulence in *Mycobacterium tuberculosis* (*M.tb*) is crucial (2). The *M.tb* contains genes encoding for methyltransferases, which constitute 3% of its genome(3). This suggests that the methylation has important biological roles in *M.tb* (4–6).

Drug repurposing is a promising approach in developing novel treatments for diseases, as it avoids the time-consuming process of developing new drugs from scratch (6, 7). Existing drugs with known safety and bioavailability profiles can be repurposed for other uses with less risk and time than developing new drugs (8). The Food and Drug Administration (FDA) only recently approved three new drugs: bedaquiline, delamanid, and pretomanid for TB (9, 10). This highlights the fall in development of novel antibiotics. Thus, drugs that interact with disease-relevant targets are being repurposed to combat TB, opening the door for more effective and precise treatments. Biologically relevant protein experiences conformational changes upon ligand binding. These ligand-induced conformational changes are often specific and depend on the drug molecule’s structure, functional groups, and binding location on the protein (11, 12).

ThyX (*Rv2754c*), flavin-dependent thymidylate synthase, has been used as a potential target for drug repurposing (13). In *M.tb, de novo* synthesis of dTMP (2-deoxythymidine-5-monophosphate) depends on two different thymidylate synthase (TS), i.e., ThyA and ThyX (14). All living things require thymidylate (dTMP) for the synthesis of dTTP (precursor of DNA), which is necessary for DNA replication and RNA maturation. The production of dTMP by cells can occur either spontaneously from dUMP or by thymidine synthase. The TS enzyme is necessary for the *de novo* synthesis of dTMP production because it adds a methyl group on dUMP at position 5 of pyrimidine ring (15).

FAD (flavin adenine dinucleotide) is tightly linked by a unique fold and is found in the ThyX family of TS (16). During catalysis, FAD facilitates the transfer of hydrides from NADPH (15, 17). Therefore, in the reaction catalyzed by ThyX, it uses FAD and NADPH as cofactors to produce reduced tetrahydrofolate (H_4_-folate, THF) from methylene-tetrahydrofolate (CH_3_H_2_-folate) rather than oxidized dihydrofolate (H_2_-folate) as in the case of ThyA which uses CH_3_H_2_-folate as a reductant and as a one-carbon source (13). A recent study using high performance liquid chromatography (HPLC) has successfully validated the prediction that *Chlamydia trachomatis* ThyX produces THF (18).

Additionally, ThyX is an essential gene for *M.tb* growth and is found to be upregulated in MDR-TB strains (19). It plays a vital role in DNA synthesis and cell survival. ThyX is rarely found in eukaryotes and absent in humans. Furthermore, ThyA is dispensable, and mycobacteria primarily rely on ThyX for thymidine, making it a promising antibacterial therapeutic target (20)(21). ThyX is found in many important human pathogens, such as *M.tb, Helicobacter pylori,* and *Bacillus anthracis*. In *M.tb*, ThyX encodes a protein of 250 amino acids and weighing 27.5 KDa. Two drugs, CGA and MSZ, were identified as potential repurposing candidates for TB using *in silico* screening (14). CGA is used to treat hyperammonemia (22), and MSZ reduces inflammation in disorders like ulcerative colitis (23).

FdUMP, a fluorinated derivative of dUMP, has been discovered to be a strong inhibitor of TS (24). The enzyme has two identical sites, and when cofactor 5,10-CH_2_H_4_ folate is present, FdUMP binds covalently to both enzymes and 5,10-CH_2_H_4_ folate (25, 26). Furthermore, binding kinetic studies with different concentrations of ligands suggests a ligand-induced sequential titration for subunit interactions, in which only one site on the free enzyme is accessible until both ligands bind, i.e., 5,10-CH_2_H_4_ folate and FdUMP, which causes conformational changes that make second internal site accessible (27, 28). The characterization of this enzyme’s three-dimensional structure will substantially aid in understanding the kinetics of ligand binding.

This study potentially provides insights into the mechanism of interaction of ligands, ligand-induced protein conformational changes, and protein stability, which are essential for identifying potential drug targets and their *in vitro* validation. Therefore, in this study, various spectroscopic techniques were used to study the interaction profile of CGA and MSZ with ThyX. The prime purpose of this work is to understand the binding kinetics, such as calculating binding constant, Gibbs free energy, and binding sites of ThyX for the drugs. The interaction study of both the drugs, CGA and MSZ, with ThyX has confirmed that the drugs cause a decrease in the intrinsic fluorescence (IF) of the protein which may potentially affect its biological activity.

## MATERIALS AND METHODS

### Materials

#### Drugs, protein purification, and estimation assembly

CGA and MSZ drugs were purchased from Cayman Chemicals and prepared in 1× phosphate-buffered saline (PBS; pH 7.4). Protein was purified from nickel-nitrilotriacetic acid (Ni-NTA; Qiagen beads). ThyX concentration was estimated using the Bradford method. A 0.22 µm Millex-LG syringe filter from Millipore (USA) was used to filter solutions. For thermal denaturation, real-time PCR was used (BIO-RAD, C1000 Touch Thermal Cycler-CFX-96). Multimode plate reader was purchased from Molecular Devices (SpectraMax M2e), SYPRO Orange and Alamar Blue (AB) from (Sigma-Aldrich).

#### Bacteria

Mycobacterial strain H37Ra was maintained in 7H9 medium with 10% oleic albumin dextrose catalase (OADC), 0.05% Tween 80, and 0.2% glycerol added as supplements. Stocks of the cultures were stored for later use in 20% glycerol at −80°C after being grown to the mid-log phase.

### Methods

#### Computational studies

FDA-approved drug library of 3,967 compounds for *in silico* screening was performed, and the two drugs with the best scores, i.e., CGA and MSZ, were chosen as potential inhibitors of ThyX and were validated by 200 ns molecular dynamic simulations of CGA and MSZ depicting effects of these drugs on ThyX’s stability, structural changes, principal component analysis, and free energy. This study was published in https://doi.org/10.1080/07391102.2021.1913230.

#### Protein expression and purification

ThyX was cloned in pET-28a and transformed in *E. coli DH5α* cloning vector, and the plasmid DNA was transformed in Rosetta *E. coli* for expression. One liter of culture was used for the induction of ThyX, and isopropyl-β-D-thiogalactopyranoside (IPTG)-induced cells were harvested using centrifugation and subsequently resuspended in a chilled phosphate-buffered saline solution containing added potassium chloride. The cells were then sonicated with regulated pulse cycles following the ice incubation period. Furthermore, the cell lysate was centrifuged to extract the ThyX protein-containing soluble fraction. An Ni-NTA column was used to purify this fraction. Elution with particular imidazole concentrations was then performed, along with several washes. Furthermore, SDS-PAGE (15%) was used to evaluate and visualize the purified fractions. Finally, a Bradford test was used to assess the protein concentration to use in the following experiments.

#### Protein drug interaction studies

##### Preparation of drug solutions

CGA and MSZ drug solutions were prepared by 1 mg of crystallized solids of drugs in 1 mL of 1× PBS buffer. The 1 mg/mL stock solution of CGA and MSZ was equivalent to 5.2 and 6.5 mM, respectively, and was diluted to prepare the desired concentration of drugs.

##### Tryptophan intrinsic fluorescence

The binding of CGA and MSZ to *M.tb* ThyX was studied using fluorescence quenching titration method. Incrementing volume (0–25 µL) of CGA and MSZ (stock concentration 250 µM) was added to protein sample (5 µM) with final volume being 500 µL. To study the fluorescence quenching mechanism of ThyX, the emission fluorescence between 300 and 450 nm was recorded after exciting the samples at 280 nm in a quartz cuvette with having a path length of 10 mm. All fluorescence data were subtracted from their respective blanks, and fluorescence measurements were adjusted for the inner filter effect. SpectraMax (M2^e^) from Molecular Devices was used to take the fluorescence spectra.

### Stability assay

#### Differential scanning fluorimetry pattern of ThyX

Differential scanning fluorimetry (DSF) is used for temperature-induced thermal stability of a protein by measuring the fluorescence quantum yield that results from a non-polar fluoroprobe that binds to the hydrophobic core of the protein, which is visible at higher temperatures. DSF is a gold-standard method for screening small compounds against protein of interest from a large ligand library due to its high-throughput nature. The reactions were incubated in 96-well plates with a final volume being 50 µL (per sample well) and were prepared using 5× SYPRO Orange dye and fixed concentration of ThyX (5 µM). An increasing concentration of CGA and MSZ (5–25 μM) was added with protein and sealed. In the absence of fluorescence, buffer was used as a blank control, while drugs without ThyX served as non-specific fluoroprobe binder controls. With an excitation at 490 nm and emission at 575 nm, the reaction was heated from 25°C to 95°C in increments of 1°C/10 s.

#### Urea-based chemical denaturation

Using urea-induced denaturation, effects of drugs on the stability of ThyX were studied. The total volume of 500 µL ThyX (5 µM) reaction solutions was prepared using a 0.5 M increasing gradient of denaturant concentration (0–8 M). After incubating the samples for 16 hours at RT, their intrinsic fluorescence was examined. Emission spectra were recorded from 300 to 400 nm to monitor the unfolding process with excitation at 280 nm. Additionally, data were plotted for fraction denatured (*f*_*D*_) and evaluated for λ_max_.

#### ANS binding assay

ThyX, ThyX-CGA, and ThyX-MSZ complexes were incubated with hydrophobic fluorescent dye ANS (aniline-8-naphthalenesulfonate) to analyze the intermediate conformational states of ThyX and drugs. ANS stock of 3 mg/mL (10 mM) was prepared in a phosphate buffer (pH 6.5). Incubation for ~20 minutes at RT was carried out, maintaining dark conditions followed by gentle mixing. The increasing concentrations of the urea denaturant were added to the protein samples with final ANS concentration of 20 µM. The samples were excited at 370 nm, and the emission spectra were recorded from 400 to 600 nm, with a step size of 5 nm in a cuvette of path length of 1 cm. All the values were normalized by subtracting the baseline value.

### Conformational studies

#### Circular dichroism spectroscopy

Circular dichroism (CD) spectroscopy is the most effective technique to study the secondary structural changes in proteins (29). To check drug-induced changes in secondary structure of ThyX, far UV-CD was performed using a Photophysics Chirascan CD spectrophotometer (United Kingdom) with 1 mm path length of the cell at RT. The far UV-CD spectra of ThyX in the absence and presence of both ligands, i.e., CGA and MSZ, were recorded from 200 to 260 nm with ThyX concentration being 5 µM. Final spectra were plotted by taking the average of triplicate samples, and data were standardized to limit baseline contribution by the buffer system.

### Anti-mycobacterial activity and drug cytotoxicity tests

#### Growth conditions of bacterial strain and cell line

*M.tb* H37Ra (ATCC 25177) was maintained in supplemented Middlebrook, 7H9 liquid broth at 37°C for drug susceptibility testing. RAW macrophages were maintained in Dulbecco's modified Eagle medium (DMEM) media supplemented with 10% fetal bovine serum and antibiotic solution.

#### Determination of minimum inhibitory concentration of drugs

For assessing the minimum inhibitory concentration (MIC) against drugs, CGA and MSZ were prepared in 1× PBS and were serially diluted in 7H9 media. The drugs were subsequently diluted up to twofold in 100 µL of 7H9 and added to the *M.tb* H37Ra culture (OD_600_ ∼0.4), inoculated in 96-well plates. The only media and cells containing wells were used as negative and positive controls, respectively. The plates were incubated at 37°C in an incubator for six continuous days. After incubation, AB solution (0.02%) was added to each well, and the plate was incubated for another 24 hours at 37°C. The change in the color by the reduction of oxidized blue resazurin to a pink resorufin was assessed.

#### *In vitro* growth curve of CGA and MSZ

The log phase cultures (OD_600_ of 0.8–1.0) of *H37Ra* were grown. Cultures were inoculated at 0.1 OD. Control cultures and treated cultures were observed by measuring absorbance using a spectrophotometer with an interval of 24 hours for 5 days to check the growth pattern.

#### Cell survival assay

The direct counting of dead cells is made easier by the flow-based propidium iodide (PI) staining. A dye that binds to DNA and is membrane impermeable, PI can distinguish between dead and live cells in a population (30). The cytotoxicity of CGA and MSZ was determined by PI staining, and 0.5 × 10^6^ cells were seeded in 12-well plates. After overnight incubation, media were changed to remove non-adherent cells. Drugs containing fresh media were added into wells. After 48 hours, media were removed, and cells were washed with fluorescence-activated cell sorting (FACS) buffer, followed by incubation with PI prepared in 1× PBS incubated for 5 minutes. After 5 minutes, cells were washed twice with 1× PBS, scraped gently, and resuspended in FACS buffer. Acquiring was done in PE and PE Texas Red channel using FACS Canto II flow cytometer (BD Biosciences, USA) (31).

#### *Ex vivo* experiments

RAW macrophages were seeded in 12-well plates and incubated in a CO_2_ incubator at 37°C overnight. The next day in the morning, the media were changed, and the cells were infected with H37Ra. Four hours post-infection, media were removed, and cells were washed twice with 1× PBS. Drugs containing new media were added to the cells followed by incubation at a 37°C CO_2_ incubator. At different time points, media were removed, cells were washed with 1× PBS, and lysed with 0.02% SDS, and different dilutions were plated on 7H11 agar plates. Colonies were counted after 15 days to calculate CFU. Graphs were plotted using GraphPad Prism.

## RESULTS

### Biophysical studies of protein-drug interactions

#### ThyX fluorescence quenching produced by CGA and MSZ

The change in the IF pattern of proteins with respect to the addition of ligands is commonly used to examine the dynamic behavior of protein-ligand interaction and several binding properties such as binding constant and number of binding sites (32). The intrinsic fluorescence of a protein is due to aromatic amino acids, such as tryptophan, tyrosine, and phenylalanine (33). It has been reported that the quenching mechanism can be of two types in which presence of ligand can reduce intrinsic protein fluorescence by static quenching where there is a formation of the complex with the fluorophore; the second one is dynamic quenching where collisions with the fluorophores provide an alternative non-radiative path for the excited state electron to reach the ground state. Dynamic quenching reveals information related to the microenvironment of fluorophore (34), while static quenching aids in determining the protein-ligand binding constants (*K*) and the number of binding sites (*n*) (35). Static and dynamic quenching can be distinguished based on three factors. First, static quenching shifts the emission maximum (max), while dynamic quenching reduces fluorescence intensity. Second, dynamic quenching increases with temperature in contrast to static quenching’s decline (36). Third, 10^10^ M^−1^s^−1^ is the bimolecular rate constant (*K*_*q*_) cut-off value for dynamic quenching. Any *K*_*q*_ value greater than 10^10^ M^−1^ s^−1^ is indicative of static quenching (37).

The fluorescence emission spectra of native ThyX exhibit a strong peak at 325 nm (λ_max_) when excited at 280 nm in the scanning range between 300 and 400 nm. Strong IF quenching with a red shift of 4–5 nm was observed with the sequential addition of CGA and MSZ to their final concentrations of 25 µM. Furthermore, fluorescence quenching data were analyzed by using the Stern-Volmer equation (1).


(1) $$\frac{F_{0}}{F}=1+K_{SV}\left[Q\right]=1+k_{q}\tau_{0}[Q]$$

where *F*_0_ and *F* represent the fluorescence intensity in the absence and presence of the drug. *K*_sv_, *K*_*q*_, and *t*_0_ are the Stern-Volmer quenching constant, bimolecular collision rate constants, and average lifetimes of biomolecules, respectively (38).

**Fig 1:**
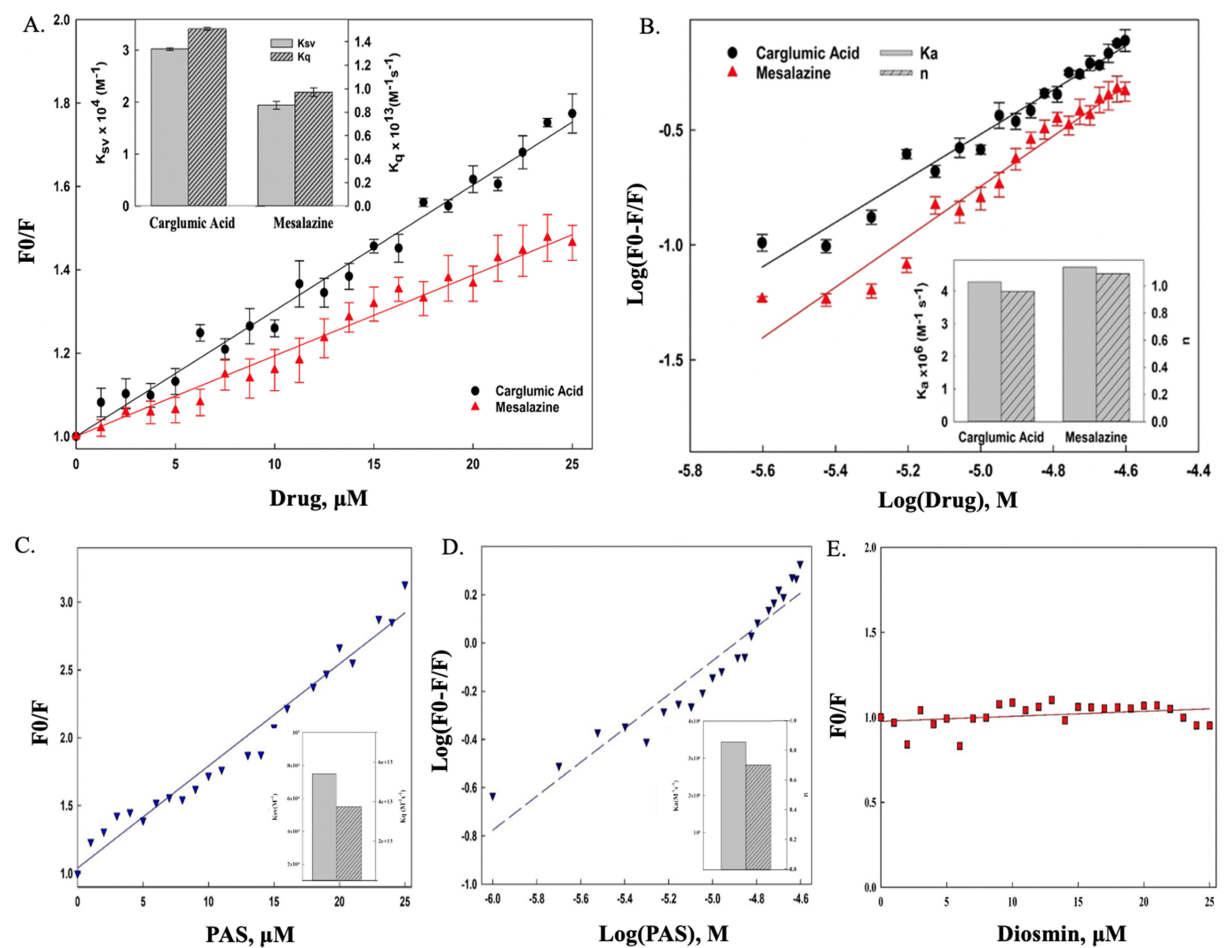
(**A**) The CGA- and MSZ-induced IF quenching of ThyX at 303.15 K. The inset shows the Stern-Volmer (*K*_SV_) constants and quenching rate constants (*k*_*q*_) of the protein-ligand interaction. *K*_SV_ values were determined by linear fitting of the CGA and MSZ binding isotherms according to equation (1). (**B**) Binding and thermodynamic parameter determination. The plot of log [(*F*_0_ − *F*)/*F*] vs log (drugs in *M*) for ThyX with CGA and MSZ system at 303 K temperature. Using the method outlined in the Results, ligand binding isotherms are nonlinearly fitted. The association constant (*K*_*a*_) and binding stoichiometry (*N*) for CGA and MSZ with respect to the protein at 303 K are shown in the inset. (**C**) The para-amino salicylic acid (PAS; positive control)-induced IF quenching of ThyX at 303.15 K. The inset shows the Stern-Volmer (*K*_SV_) constants and quenching rate constants (*k*_*q*_) of the protein-ligand interaction. *K*_SV_ values were determined by linear fitting of the PAS binding isotherms according to equation (1). (**D**) Binding and thermodynamic parameter determination. The plot of log [(*F*_0_ − *F*)/*F*] vs log (drugs in *M*) for ThyX with PAS system at 303 K temperature. Using the method outlined in the Results, ligand binding isotherms are nonlinearly fitted. The association constant (*K*_*a*_) and binding stoichiometry (*N*) for PAS with respect to the protein at 303 K are shown in the inset. (**E**) Stern-Volmer plot showing no interaction between ThyX and diosmin (negative control). Since the plot fit is non-linear, the binding parameters could not be calculated.


The linear fitting of plots of *F*_0_/*F* vs drug yielded the slope utilized as Stern-Volmer quenching constant (*K*_sv_). The linear Stern-Vomer plot revealed that the strong fluorescence quenching (*K*_sv_), along with a slight red shift in λ_max_ of ThyX with respect to the addition of the quenchers, indicates that both drugs strongly bind to the ThyX and result in fluorescence quenching by either exposing the tryptophan residues to the slight polar environment but mainly through direct interaction with them. Furthermore, the collision constant of >10^12^ indicates that the fluorescence quenching of ThyX is the consequence of static quenching. This suggested the formation of a drug-protein complex (Fig. 1A). Furthermore, fluorescence emission data were used to determine binding constant (*K*_*a*_) and the number of binding sites (*n*) of ThyX with both drugs employing the modified Stern-Volmer equation (2) (39).

(2) $$log\left(\frac{F_{0}-F}{F}\right)=logK_{a}+nlog\left[Q\right]$$

where the intercept of the equation represents the binding constant (*K*_*a*_), while the slope represents the number of binding sites (*n*). The binding constant of ThyX with both drugs was found to be in the magnitude of *K*_*a*_ ≅ 10^6^ M^−1^, which shows that both drugs bind strongly to their target and will have a strong inhibitory effect through binding in a stoichiometry of ~1:1 (Fig. 1B).

To validate our binding assay, we performed tryptophan fluorescence quenching experiments with positive and negative controls. For positive control, PAS was selected, which is an important second-line anti-tuberculosis drug and is known to bind to multiple components of the folate cycle, including ThyA and ThyX proteins. Studies using differential scanning calorimetry have demonstrated that PAS stabilizes ThyX, confirming its binding affinity (40). For calculating binding parameters, PAS was sequentially titrated against ThyX, and the resulting Stern-Volmer analysis yielded a quenching constant (*K*_SV_) of 7.5 × 10⁴ M⁻¹ and a bimolecular quenching rate constant (*k*_*q*_) of 3.75 × 10¹³ M⁻¹s⁻¹. Because the *K*_*q*_ value is several orders of magnitude greater than the diffusion-controlled limit (10¹⁰ M⁻¹s⁻¹), these results confirm a static quenching mechanism stabilizing ThyX-PAS complex. Furthermore, *K*_*a*_ (binding constant) and *n* (binding sites) were calculated using equation (2). It was found that the PAS-ThyX complex has a *K*_*a*_ value of 3.44 × 10^6^ M^−1^s^−1^ and *n* ⁓1. The results are detailed in Fig. 1C and D; Table 1. PAS binds strongly with ThyX, with a ΔG value of −37.91 kJ/mol.

**TABLE 1:** Values of binding and thermodynamics parameters of CGA, MSZ, and PAS with ThyX at 303 K

Drugs	*K*_*a*_ (× 10^6^ mol^−1^)	*K*_*q*_ (× 10^13^ mol^−1^ s^−1^)	*K*_sv_ (× 10^4^ mol^−1^)	Δ*G* (kJmol^−1^)
CGA	4.28	1.5	3.02	−38.09
MSZ	4.73	9.7	1.94	−38.33
PAS	3.44	3.75	7.5	−37.91

For a negative control, we used diosmin (a citrus flavonoid glycoside with no reported anti-mycobacterial activity). Titration of ThyX with diosmin resulted in no significant change in tryptophan fluorescence. Consequently, the Stern-Volmer plot was scattered and showed no valuable trend, confirming a lack of interaction and precluding the calculation of binding parameters (Fig. 1E).

### Studies on stability

#### Thermal shift assay and differential scanning fluorimetry

The thermal shift assay was performed to understand the effect of both drugs on the stability of ThyX. ThyX was sequentially heated from 25°C to 95°C with a step size of 1°C for a duration of 30 s in the presence of non-polar fluorophore SYPRO Orange exhibits a thermal melt profile between 40°C and 60°C. The raw melt curve data were resolved by differential equation (3).


 (3) FD=-(dRFU)/dT

**Fig 2:**
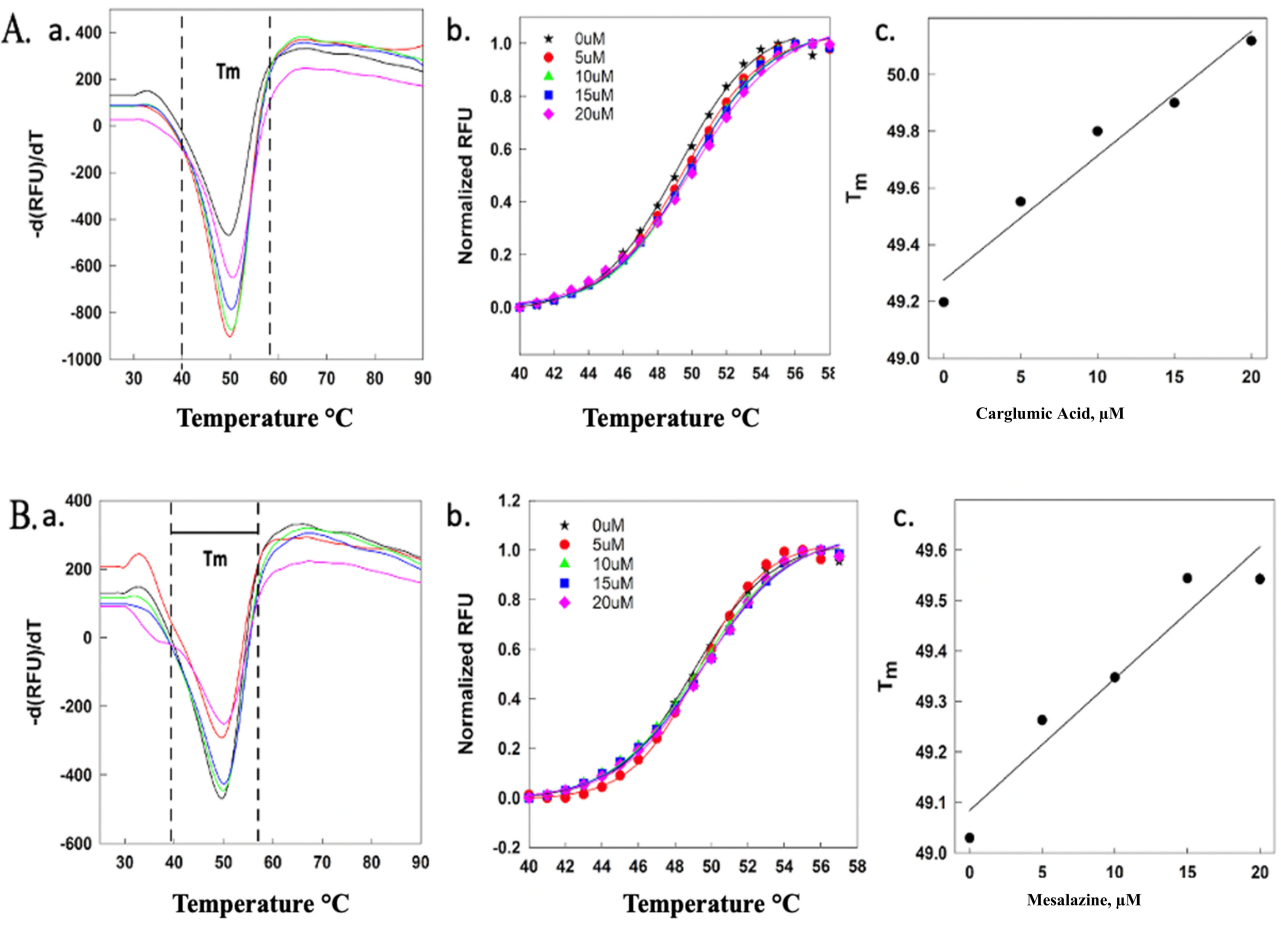
Differential scanning fluorimetry of CGA (**A**) and MSZ (**B**) represents the first derivative of the melt curve (**a**), normalized fluorescence (**b**), and melting temperature difference Δ*T*_*m*_ of the drugs (**c**).


In the presence of increasing concentrations of CGA and MSZ (5–20 μM), the thermal profile of ThyX increased by 1°C and 0.5°C, respectively (Fig. 2A and B). The data from the thermal melt assay revealed that both drugs have a slight stabilizing effect on ThyX, which can be the direct consequence of stable complex formation. The data from this thermal shift assay are in good agreement with our fluorescence quenching study.

 (4) $$Y=\frac{{(F}_{min}-F_{max)}}{1+e^{\frac{\left(T-Tm\right)}{m}}}+F_{max}$$

From the above Boltzmann equation, the slope within the transition range of *T*_*m*_ from the maximum of the first derivative using equation (4) was used to determine the melting transition window of 40°C–58°C (Fig. 2A and B). *F*_max_ and *F*_min_ are the maximum and minimum fluorescence intensities, respectively, and *m* is the slope within the transition range of *T*_*m*_. Equation (5) was used to further normalize the data to a scale of 0–1 (Fig. 2Ab and Bb).


(5) $$F_{n}=\frac{F_{i}-F_{min}}{F_{max}-F_{min}}$$

The maximum concentrations of CGA and MSZ were found to cause a slight increase in the melting temperature (Δ*T*_*m*_) (Fig. 2Ac and Bc).

#### Drug-induced stabilization/destabilization of ThyX in the presence of chemical denaturants

**Fig 3:**
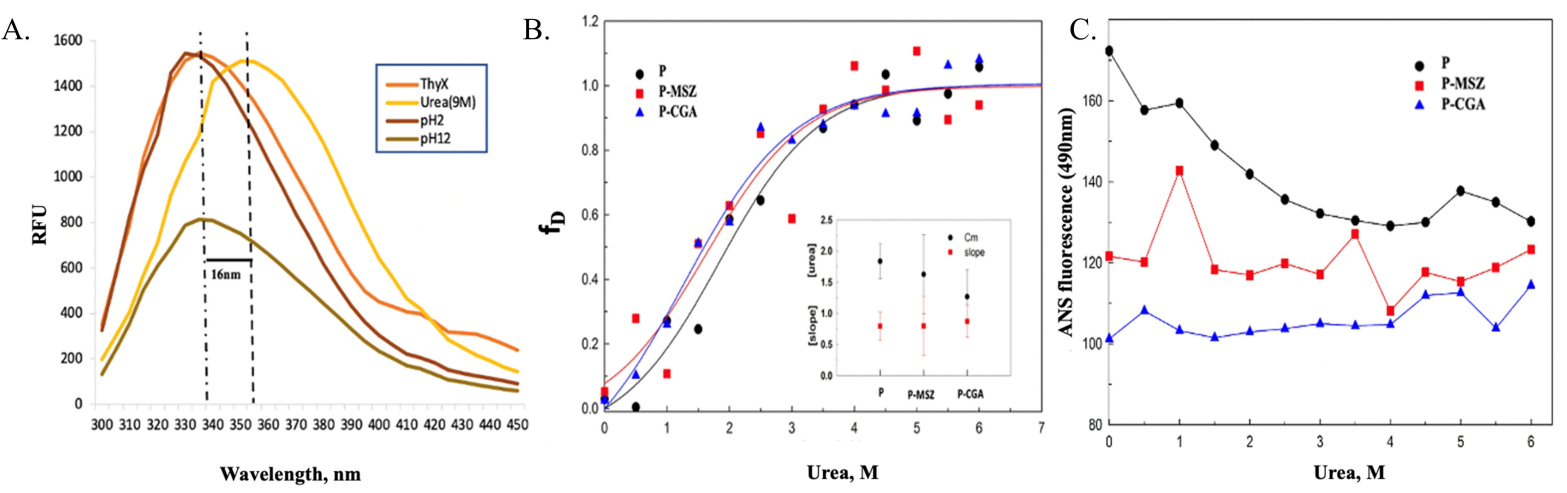
Chemical denaturation of ThyX. (**A**) Screening of denaturation buffers for ThyX, where no significant unfolding is seen in pH 2 (low pH) and pH 12 (high pH) but urea induces unfolding. (**B**) Urea-induced chemical denaturation of ThyX complexes with CGA and MSZ, with midpoint and slope of the fraction denatured displayed in the inset (**C**). ANS observed (at 490 nm) urea-induced unfolding curves of ThyX in the absence and presence of CGA and MSZ.

Denaturants, including urea, high pH (pH 12 glycine buffer), and low pH (pH 2 glycine buffer), were used in the initial screening process to determine the best chemical denaturant for investigating the unfolding of proteins. Initially, these denaturants were screened using a 5 µM protein sample and a control group with only a diluted protein sample containing only buffer (1× PBS). When the 300–450 nm spectra were analyzed, it was found that while both high and low pH did not affect the unfolding process, urea significantly increased the amount of protein unfolding when compared to the control (Fig. 3A).

To further validate the stabilizing effect of both drugs on ThyX, established through thermal shift assay, urea-based denaturation of ThyX alone and in the presence of drugs (10 µM) was carried out. As mentioned in the section ThyX fluorescence quenching produced by CGA and MSZ, ThyX exhibits a strong fluorescence from 300 to 400 nm with a wavelength maximum of 325 nm. The addition of increasing concentration of urea caused a sequential increase in the λ_max_ with a large redshift of 20–25 nm indicating strong denaturation of ThyX (Fig. 3B). The data for λ_max_ were further resolved and plotted for fraction denaturation using equation (3).


(3) $$f_{D}=\frac{F-Fd}{Fn-Fd}$$

where *F* is the maximum fluorescence intensity, while *F*_*n*_ and *F*_*d*_ are the maximum fluorescence intensity of native and denatured ThyX. The fraction denaturation plot revealed that the presence of both CGA and MSZ caused an increase in denaturation rate (denaturation slope) and a decrease in (denaturant) midpoint, which again points toward the stabilizing effect of both drugs on ThyX, which again validates our DSF result (Fig. 3B inset).

#### ANS assay

The denatured samples and control after incubation were studied by ANS assay. As protein unfolds at increasing urea concentration, ANS fluorescence decreases (Fig. 3C). This suggests that urea-induced unfolding of ThyX disrupts its surface hydrophobicity in the native state as well as hydrophobic clusters upon denaturation. The binding of CGA and MSZ in the absence of urea further decreases ANS hydrophobicity. The drugs cover or mask the surface hydrophobic nature of ThyX. Therefore, the urea unfolding of the drug-protein complex does not produce any change in the ANS fluorescence.

### Conformational-based study

#### Circular dichroism

**Fig 4:**
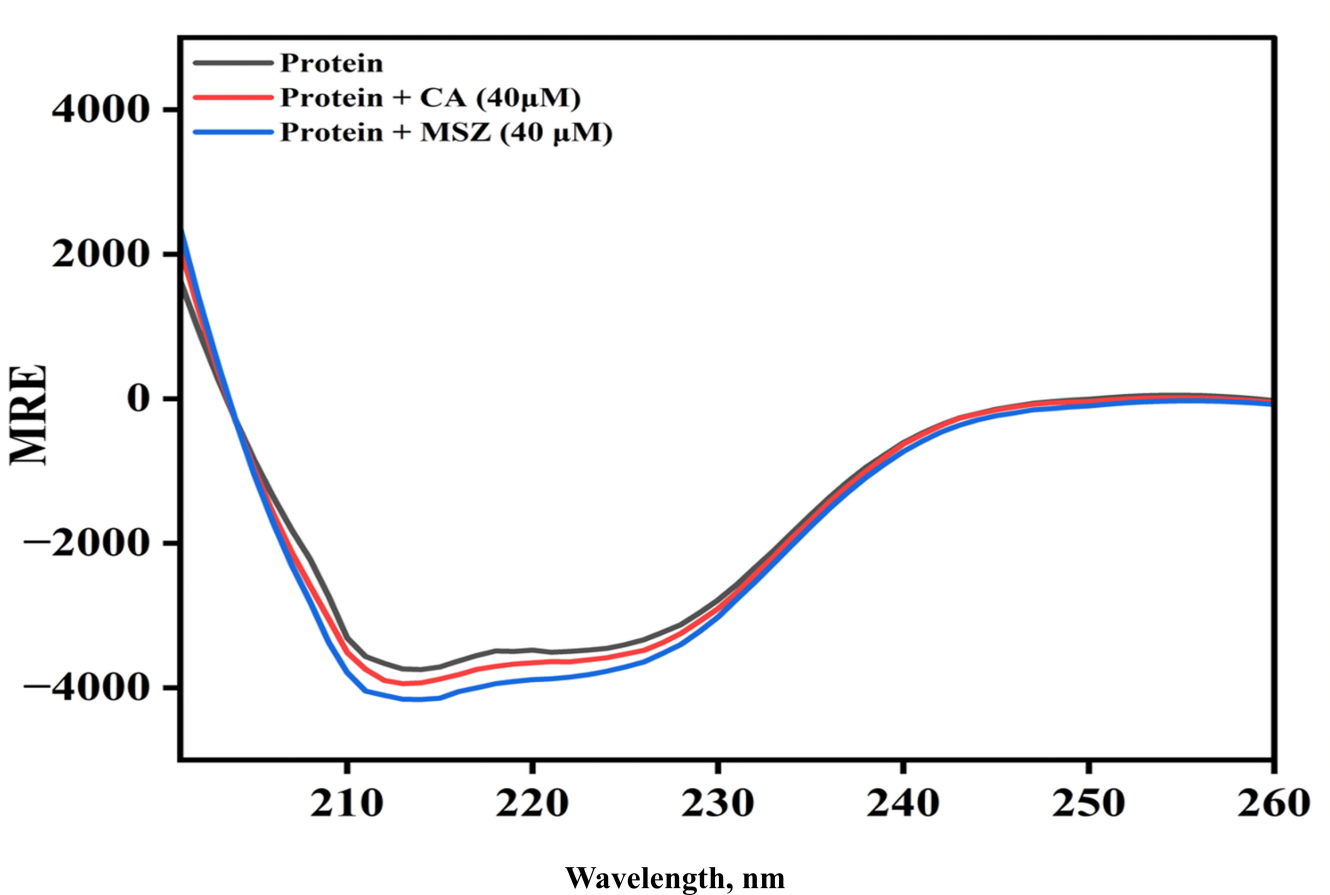
Far UV-CD spectra of ThyX in the absence and presence of CGA and MSZ. Effect of CGA and MSZ on the secondary structure of ThyX at pH 7.4. Each spectrum is the average of four separate spectra, and each spectrum’s background is removed using the appropriate blank.


Far UV-CD spectroscopy is a widely accepted technique to determine the secondary structural features of proteins. To determine the effect of drugs on the secondary structure of ThyX protein, far UV-CD of ThyX (5 µM) in the absence and presence of drugs (MSZ and CGA) was measured. The protein spectra were scanned from 200 to 260 nm at 40 µm concentration of MSZ and CGA. The CD spectra of ThyX peaked at 218 and 222 nm, characteristic α-helix in the protein. The negative band appears in the spectra at 218 and 222 nm and is due to *n*→π* and π→π* transitions. Upon addition of drug concentration, the negative ellipticity of ThyX decreased, revealing the interaction of MSZ and CGA with protein and increasing the secondary structure contents of the protein (Fig. 4). The data were converted to mean residual ellipticity (MRE) according to equation (4).

(4) $${\left[\theta \right]}_{222}=\frac{M_{o}\theta_{\lambda }}{10lC}$$

(5) $$\alpha \text{-helix }(\%)=\frac{-\left[MRE_{222}-2340\right]}{\left[30300\right]}\times 100$$

where *M*_*o*_, θ_λ_, *C,* and *l* are, respectively, the mean residual weight of the ThyX, observed ellipticity in millidegree at wavelength, protein concentration in mg/mL, and path length of the cell, respectively, MRE_222_ observed ellipticity at 222 nm.

Furthermore, the % α-helical content of ThyX in the absence and presence of CGA and MSZ was determined by calculating MRE values according to equation (5). The α-helical contents were calculated and found to be 19.20%, 19.98%, and 20.55% in ThyX, GCA-ThyX, and MSZ-ThyX, respectively. Upon the addition of GCA and MSZ, the α-helical content of ThyX increased, suggesting the more compact structure of protein after the interaction of ligands.

### Evaluating drug cytotoxicity and anti-mycobacterial efficacy

#### CGA and MSZ showed anti-mycobacterial activity in *in vitro* conditions

**Fig 5:**
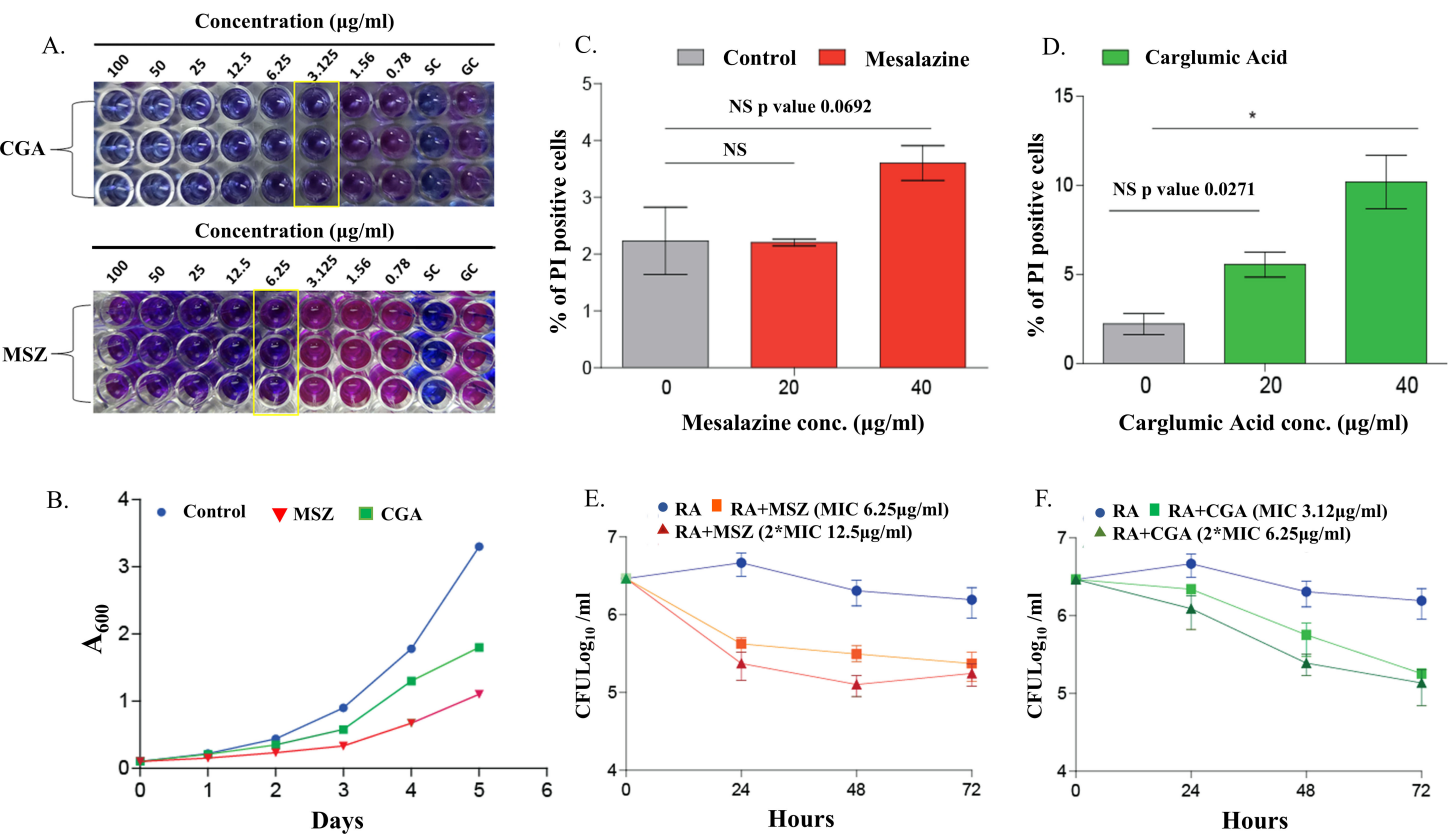
(**A**) MIC determination of CGA and MSZ against drug-sensitive *M.tb* H37Ra. Negative and positive controls were used as sterile control and growth control, respectively. (**B**) H37Ra cultures were grown to an A_600_ of 0.8 and used to seed the fresh cultures at an initial A_600_ of 0.1; day-wise growth was observed for five continuous days, and the graph was plotted. (**C and D**) RAW macrophages were cultured for 48 hours in DMEM medium with different drug concentrations of CGA and MSZ, followed by PI staining and flow cytometry analysis. (**E and F**) RAW macrophages were infected with H37Ra, and different concentrations of drug treatment were given for MSZ (6.25 and 12.5 µg/mL) and CGA (3.12 and 6.25 µg/mL) after termination of infection at 4 hours. CFUs were enumerated at different time points 24, 48, and 72 hpi. One-way ANOVA was used to check the statistical significance in GraphPad Prism software.

Next, to test the efficacy of the MSZ and CGA against *M.tb,* we performed MIC experiments using the Alamar Blue assay. The lowest concentration of the drug which effectively inhibited the growth of *M.tb* was considered as the MIC of the drug. Here, we have found that CGA and MSZ both inhibit mycobacterial growth at 3.12 and 6.25 µg/mL, respectively (Fig. 5A). Furthermore, we have performed the absorbance-based analysis to check the inhibitory effects of drugs on mycobacterial growth by using MIC concentrations. For this *M.tb H37RA,* cultures were inoculated at 0.1 OD, and drugs were added in independent cultures in biological triplicates. No drug was used as a control. Control and drug-treated cultures were observed through absorbance at 600 nm with an interval of 24 hours for 5 days. Here, we have observed the slow growth of bacteria in the presence of MSZ and CGA compared to the control (Fig. 5B). These results suggest that MSZ and CGA have the potential to restrict *M.tb* growth under *in vitro* conditions.

#### CGA and MSZ showed anti-mycobacterial activity in *ex vivo* conditions

The above experiments demonstrate the potential of selected drugs in *in vitro* growth conditions. Next, we wanted to check the effects of these drugs on the mycobacterial survival inside the host. For that purpose, the RAW macrophage cell line was used. First, we have performed the cell survival assay to check cell viability in the presence of drugs MSZ and CGA using PI staining by FACS analysis. Here, we have not observed any significant cell death in the presence of MSZ up to 40 µg/mL concentration, while CGA showed safe use till 20 µg/mL since some death was observed at 40 µg/mL (Fig. 5C and D). Subsequently, we did *ex viv*o CFU analysis with two concentrations of the drug, one at MIC and 2× MIC; for MSZ, MIC is 6.25 µg/mL, and 2× MIC is 12.5 µg/mL, and for CGA, MIC is 3.12 µg/mL, and 2× MIC is 6.25 µg/mL. For this, RAW macrophages were seeded in 12-well plates overnight, and cells were infected at 1:10 MOI with H37RA. After 4 hours, the infection was terminated by removing the media, washing two times with 1× PBS, and adding fresh media containing different concentrations of MSZ (6.25 and 12.5 µg/mL) and CGA (3.12 and 6.25 µg/mL) in different wells. For CFU analysis, plating was done at different time points 24, 48, and 72 hpi on 7H11 plates. Colonies were counted after 15 days. No drug-added wells were used as a control. Here, we have observed a significant change in bacterial survival in the presence of MSZ and CGA at both concentrations (Fig. 5E and F). Together, these results reveal the potent anti-mycobacterial activity of MSZ and CGA. This positions them as promising candidates for drug repurposing, and further investigations focused on host interactions. This experiment also demonstrates the potential of MSZ and CGA to be further investigated in *in vivo* studies.

## DISCUSSION

In recent years, significant advancements in TB therapy have been made, and new candidates have been added to the pipeline by drug repurposing. Drug discovery for TB is a challenging process since trials are lengthy and intricate. Therefore, to overcome the emergence of drug-resistant *M.tb* strains, it has become necessary to develop new anti-TB compounds and identify novel therapeutic targets. The ThyX protein was found during our initial search as a potential therapeutic target since its homologs are not present in humans and are crucial for *M.tb* survival, making it an incredibly effective drug target for the treatment of TB. This has led us to investigate ThyX’s potential as a target against already-approved drugs like CGA and MSZ.

In this study, multiple spectroscopic approaches were used to investigate the interaction between ThyX and ThyX-drug complexes (CGA and MSZ). Our investigation reveals that CGA and MSZ bind with ThyX and cause reduction of intrinsic fluorescence majorly through static quenching process. ThyX shows fluorescence between 300 and 400 nm with the emission peak (max) occurring at 325 nm when excited at 280 nm. ThyX fluorophore intensity steadily declines, with a little red shift of 10 and 5 nm for CGA and MSZ, respectively. The binding investigations indicate that ThyX binds to CGA and MSZ with an affinity of *K*_*a*_ 10^6^ M^−1^ and that H-bonding, water bridges, and hydrophobic interaction stabilize the complex. The number of binding sites is almost equal to 1 in the case for both drugs.

The tertiary structure of ThyX goes through a cooperative thermal unfolding transition with a thermal melt profile between 40°C and 60°C for both CGA and MSZ. To analyze the ThyX protein unfolding patterns in the presence or absence of MSZ and CGA, urea-induced denaturation was carried out. As urea was added in increasing concentrations, it led to a sequential drop in fluorescence and a significant red shift of 20–25 nm, which indicated that ThyX had been strongly denatured. To further examine the intermediate conformational states of ThyX and drugs, binding of the hydrophobic fluorescent dye ANS with ThyX, ThyX-CGA, and ThyX-MSZ was performed that reflected that even in the absence of urea, CGA and MSZ binding further reduces ANS hydrophobicity. Hence, urea unfolding of the drug-protein complex does not produce any change in the ANS fluorescence. The CD results suggested CGA and MSZ induce conformational change in ThyX. It may be concluded from the abovementioned investigations on binding, conformation, and thermal stability that CGA and MSZ have a considerable affinity for ThyX, one of the important proteins of *M.tb*, and can potentially affect its activity.

CGA and MSZ also showed *in vitro* anti-mycobacterial activity. First, MIC was performed for CGA and MSZ, and the MIC values were found to be 3.12 and 6.25 µg/mL, respectively, in *M.tb* H37Rv cultures. We also assessed the growth pattern of *M.tb* in the presence and absence of drugs consecutively for 5 days where a significant decrease in the growth rate of *M.tb* was observed in the presence of drugs in both *in vitro* and *ex vivo* conditions. In the light of binding, conformational, and thermal stability studies followed by *in vitro* and *ex vivo* anti-mycobacterial studies, as mentioned under section Evaluating drug cytotoxicity and anti-mycobacterial efficacy , it can be concluded that CGA and MSZ could be a promising therapeutic candidate for the treatment of TB.

In recent years, drug repurposing has emerged as a promising strategy, particularly for identifying new treatments for *M.tb*. Numerous researchers have investigated the anti-mycobacterial potential of various drugs and natural compounds by integrating computational methods with *in vitro* validation. Similarly, various groups have targeted ThyX as a potential drug target and reported several potential inhibitors. In one study using chemoinformatic and *in vitro* screening, 2-chloro-3-(4-methanesulfonylpiperazin-1-yl)-1,4-dihydronaphthalene-1,4-dione and idebenone were identified as inhibitors of *M.tb* ThyX (41). Another group identified that plumbagin (extracted from plant *Plumbagin indica*) causes significant inhibition on ThyX and, in turn, shows promising anti-mycobacterial activity (42). Additionally, studies from other groups have reported the efficacy of 5-(3-octanamidoprop-1yn-1yl)-2′-deoxyuridine-5′-monophosphate as an inhibitor for ThyX (43). Similarly, several studies have been published exploring various targets of folate biosynthesis like Rv2671 and its functional analog di-hydro folate reductase, and compound para-amino salicylic acid is now an established inhibitor of multiple targets of folate cycle (40, 44). These findings, along with numerous other studies, depict the potential of repurposed drugs as effective therapeutics for TB.
